# Experimental and visual research on the microbial induced carbonate precipitation by *Pseudomonas aeruginosa*

**DOI:** 10.1186/s13568-017-0358-5

**Published:** 2017-03-09

**Authors:** Yang Bai, Xu-jing Guo, Yun-zhen Li, Tao Huang

**Affiliations:** 1 College of Geosciences and Environmental Engineering, Southwest Jiaotong University, X4434, Chengdu, 611756 Sichuan China; 2 College of Resources and Environment, Chengdu University of Information Technology, Chengdu, 610225 China; 3 Sichuan Academy of Environmental Science, Chengdu, 610041 China

**Keywords:** MICP, Calcium carbonate, Biomineralization, Biofilms

## Abstract

Microbial induced carbonate precipitation (MICP) is a common occurrence of geochemistry influences in many fields, such as biological, geographical, and engineering systems. However, the processes that control interactions between carbonate biomineralization and biofilm properties are poorly understood. We develop a method for real time, in situ and nondestructive imaging with confocal scanning microscopy. This method provides a possible way to observe biomineralization process and the morphology of biomineralized deposits within biofilms. We use this method to show calcite biominerals produced by *Pseudomonas aeruginosa* biofilms which extremely change biofilm structures. The distribution of calcite precipitation produced in situ biomineralization is highly heterogeneous in biofilms and also to occur primarily on the bottom of biofilms. It is distinct from those usual expectations that mineral started to precipitate from surface of biofilm. Our results reveal that biomineralization plays a comprehensive regulation function on biofilm architecture and properties.

**Electronic supplementary material:**

The online version of this article (doi:10.1186/s13568-017-0358-5) contains supplementary material, which is available to authorized users.

## Introduction

Microbial biomineralization is a ubiquitous process in almost all kinds of natural environments (Benzerara et al. 2006; Grotzinger and Knoll 1999). A typical example of biomineralization is the formation of stromatolites in sedimentary environment as a result of microbial mediated inorganic precipitation from the ambient environment (Perri and Spadafora 2011). Biomineralization is also involved in many anthropogenic processes, leading to unfavorable consequences that either reduce the effectiveness of engineering approaches or cause adverse effects. Biomineralization induced membrane clogging could reduce the performance of water filtration in a wastewater treatment plant. Presence of mineralized biofilms in medical devices, such as catheter, lead to serious infections of patients (Stickler 2008; Warren 2001; Jacobsen et al. 2008). Many studies have reported that at least 200 kinds of bacteria were involved in calcium carbonate biomineralization (Li et al. 2015), including *Pseudmonas*, *Sporosarcina*, *Azotobacer*, etc. Microorganisms induced carbonate precipitation by a variety of microbial metabolisms, such as photosynthesis, urea hydrolysis and nitrification, which dramatically changes saturation index (SI) of calcium carbonate. Microbial metabolism alters chemical compositions within the biofilm and in ambient environments, leading to conditions that favor the precipitation of carbonate minerals. While these biogeochemical processes have been thoroughly studied at macro scale (Lian et al. 2006), the details that how bacteria and microbial activities are involved in the mineralization processes are still poorly known (Reid et al. 2000).

Biomineralization usually appears in biofilms, which has heterogeneous structure with aggregation of surface attached microbial cells (Shiraishi et al. 2008; Wimpenny et al. 2000). With its special structure and characteristics, the interplay between biofilms and local chemical environment was shown up and make highly diverse conditions inside and outside of biofilms (De Beer et al. 1994; Ramsing et al. 1993). The activities occurred in biofilms, such as cellular respiration and amino acid sequence reveal the spatial variation of oxygen utilization. Previous studies believed that the extracellular polymeric substance (EPS) formed by biofilms was a crucial consideration for investigating microbes physiological functions and activities (Giuffre et al. 2013; Braissant et al. 2003). These ex situ research also found EPS could regulate spatial position of precipitation while mineralization. In absence of microscale chemical gradients, nucleation models estimate that the crystals produced by biofilms distribute randomly in EPS (Arp et al. 2001). Most current studies of biofilms and biomineralization suggest that precipitation of mineral appears on the biofilm surface primarily (Zhang and Klapper 2010). However, these studies and models are not suitable for in situ observation as lacking information of mineral formation in biofilms, such as spatial patterns. Accordingly, the regulation mechanisms of original precipitation and entire accumulation of mineral deposits are still not understood.

A few well designed flow cells were utilized to cultivate organisms which make it possible to control certain conditions and probe into the biofilms growth. Drip-flow reactors support a comfortable place for culturing biofilms in free-surface and slow flow conditions (Goeres et al. 2009). Even through this system was suitable for microsensor monitoring, it can’t serve in situ imaging, as microbial colony grown on the glass slides should be removed from the system and check. More recently, the interactions between heterogeneous biofilms and complex environment has propelled several flow cell designs to develop for biofilm growth observation in controlled chemical conditions. The effect of bulk fluid flow on biofilm growth has been observed in circular pipes (Horn et al. 2003), semicircular pipes (Teodósio et al. 2011), square or rectangular channels (Pereira et al. 2002; Stoodley et al. 2002). Ever since the microscope experiments of multi-channel flow cells were realized on stage and achieved nondestructive imaging of biofilm, this carrier made of Plexiglas had been broadly used (Christensen et al. 1999; Wolfaardt et al. 1994). A novel microfluidic flow cell provides the capability of reliable control of flow distributions and chemical gradients in biofilm studies (Song et al. 2014), and is thus adapted in the current study to investigate the biomineralization processes.

Here, based on the rather mature research methods and experiments of *Pseudomonas aeruginosa* on microbiology, this study was aimed at developing a real time, in situ and nondestructive visual method to observe and reveal the processes of calcium carbonate biomineralization and mineral formation within *P. aeruginosa* biofilms and estimate the consequent variation of biofilms properties. We utilize microfluidic flow cell as a carrier to show images and processes of mineral formation resulting from biomineralization in order to present the spatial distribution of mineral deposit and influence on the biofilm structure by detaching cells.

## Materials and methods

### Experimental systems set up

**Fig. 1:**
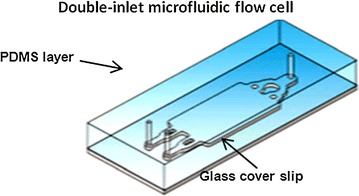
Double-inlet microfluidic flow cell design: 3D rendering of the chambers and channels (Song et al. 2014)

Experiments were carried out according to a novel designed flow cell (Fig. 1) consisted of glass cover slips and polydimethylsiloxane (PDMS) to achieve the whole processes of biofilm culture and reaction with biomineralization medium. Microbial colony growth and biomineralization processes were observed in this system, a double-inlet flow cell has two inlets for introducing two different fluids in order to achieve the chemical gradients within the flow cell under controlled conditions. The flow cells are composed of glass cover slips fixed on elastomer PDMS bodies. By the design of flow cell, the inner chambers and channels were all formed in PDMS. It supports continuous culturing of biofilms under a user-controlled growth medium, as described previously (Song et al. 2014), and glass cover slip supplies an even, strict, and transparent interface suitable for optical operation and further imaging.

### Strains and inoculation procedure

We used PAO1-*gfp* (ATCC 7700) with improved genes which expresses green fluorescent protein (GFP) in these studies (Liu et al. 2009; Zhang et al. 2011). In the natural environment, *P. aeruginosa* is a normal biofilm-forming organism which was studied as a model organism for many biofilm studies (Mann and Wozniak 2011), it is also an opportunistic pathogen (Goeres et al. 2009) which has many advantage with fine properties for experimentation. The colony was moved to 3 ml of sterile tryptic soy broth (TSB) in a culture tube and through an overnight shaking at 225 rpm and 37 °C. For the mid-log phase culture, fifty microliters of this culture was transferred to 3 ml of sterile tryptic soy broth (TSB), at 225 rpm and 37 °C, and then diluted with sterile 1% TSB to OD_600_ = 0.01 for inoculation into the flow cells. To quantify the turbidity of liquid cultures was used optical density (OD) measured at 600 nm, which was used as a corresponding measure of cell density. Under no flow (stagnant) condition, inoculated bacteria were injected into flow cell and attach to the cover slip for 1 h, and then continuously pumped into nutrient with 1% TSB medium. To avoid the disturbance by the medium, the biofilms were inoculated and cultured for 3 days to grow mature at a flow rate of 10 ml h^−1^.

### Calcium carbonate biomineralization in biofilms

The stock solutions of NaHCO_3_ (1 M) with purity of 99.5 to 100.5% (Sigma-Aldrich) and CaCl_2_ (0.5 M) (99%; Sigma-Aldrich) were sterilized with 0.2 μm cellulose acetate membrane filters for preparing. PAO1-*gfp* biofilms grown for 3 days as described previously were continuously feed with a saturated Ca(HCO_3_)_2_ solution containing 15 mM (each) CaCl_2_ and NaHCO_3_ in 1% TSB. This saturation solution was pumped into flow cells for 12–17 h at the flow rate of 10 ml h^−1^ to grow the biofilms and biominerals. As in this experiment, the calcium carbonate solution was oversaturated and 1% TSB was well diluted, the influence of organic ligands can be ignored. The SI was calculated by Eqs. 1 and 2:

1 $$IAP = [Ca^{2 + } ][CO_{3}^{2 - } ]$$

2 $$\varOmega = LAP/K_{s}$$

where [Ca^2+^] and [CO_3_^2−^] are the concentrations of Ca^2+^ and CO_3_^2−^ in solution, *K*_*S*_ is the calcite solubility product constant. pH of this solution is 7.6 and the SI logΩ = 1.88. logK_S_ = −8.46 under atmospheric partial pressure of carbon dioxide (pCO_2_) (Plummer and Busenberg 1982). All these data were measured at room temperature (22 °C). To avoid the disturbance of mineral medium on biofilm morphology (dome-shaped colonies surrounded by lawns like cells), biofilms were grown under the condition without biomineralization medium. After biofilms matured, supersaturated calcium carbonate medium were injected to stimulate biomineralization within biofilms.

### Confocal imaging

Confocal laser scanning microscopy (CLSM) (Leica TCS SP2) was utilized to perform in situ imaging with a 63× oil objective. The GFP fluorescence of biomass was excited by a 488 nm argon ion laser. Mineral precipitates was imaged by the reflection signal of excitation laser which was detected in a window between 483 and 493 nm. These two signals were recorded synchronously by CLSM. Velocity software package (PerkinElmer, Inc.) was used to visualize and quantitatively match the biofilm biomass and mineral deposits. Fiji was used for analysis of mineral particles. Firstly, binary format of the image was obtained by adjusting an adequate thresholding, and the particle areas were identified and calculated with the Particle Analysis function in Fiji. The biomass was quantitatively measured with a matlab program for analysis of biofilm spatial pattern (BioSPA).

### Confocal Raman microscopy

Confocal Raman microscopy (Princeton Instrument TriVista CRS) was utilized to determine mineral composition of biomineralized deposits. Before scanning, the flow cell was rinsed with DI water for 12 h under the same flow rate to eliminate the residual chemicals and then dried under the ambient atmosphere. The cover-glass was then gently retrieved from the flow cell and Raman scanning was performed. The CRS has a 514 nm argon ion laser, and is equipped with a 100× objective optical microscope and a liquid nitrogen cooled charge-coupled device (CCD) detector. The sample was scanned at a range of 100–1600 cm^−1^ Raman shift with a step of 1.6 cm^−1^.

### Monitoring of cell detachment during biomineralization

Colony-forming unit (CFU) counting with the effluent samples from the flow cell was performed to monitor the cell detachment over the course of experiment. To validate the sterilization of the experimental system, two effluent samples were collected at 1 and 2 h before the introduction of biomineralization media. The effluent samples were then collected at 0, 1, 3, 4, 5 and 17 h during the process of biomineralization. As the control, separate experiments were also operated in untreated biofilms. All the samples were vibrated to make cells fully dispersed before plating.

## Results

### Biomineral morphology and mineralogy analysis

**Fig. 2:**
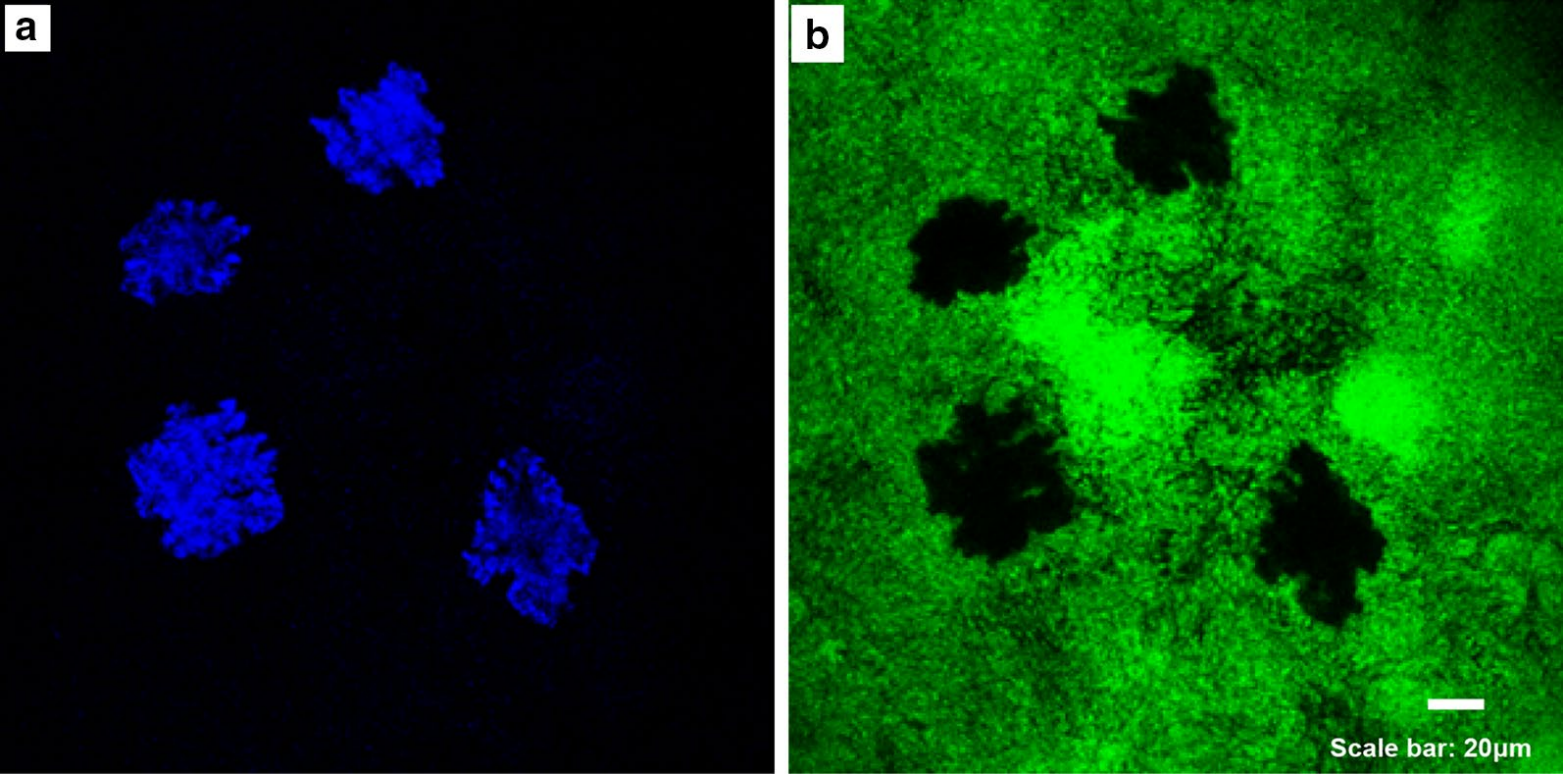
Imaging of mineral deposits in *P. aeruginosa* biofilms by confocal laser reflection. Calcium carbonate minerals and biofilm morphology were imaged in PAO1-*gfp.* **a** Minerals imaged by laser reflection show in *blue*, **b** cells show in *green* (PAO1-*gfp*)

First of all, we had confirmed that the mineral medium was nontoxic to PAO1-*gfp *cells (See Additional file 1: Fig. S1). Growth of the biofilm and formation of the mineral deposits are shown in Fig. 2. *P. aeruginosa* has been known to produce very thick and mushroom types biofilm structure. We observed both flat and mushroom type morphology during the experiment (Figs. 2b, 4). In the biomineralization experiment where biofilms were exposed to supersaturated calcium carbonate medium, the flow cell was covered with green fluorescence signal, indicating coverage of *P. aeruginosa* biofilm (Fig. 2b). However, the green fluorescens signal was characterized with four voids, which was probably attributed to the presence of precipitated mineral deposits. The laser reflectance signal was then measured with the confocal microscope to test this hypothesis. The reflection signal showed four island-like area that were significantly brighter than the rest area. More interestingly, the outlines of these blocks perfectly matched the outlines of the four voids in the green fluorescence signals, indicating the structuring of materials with different reflection properties inside the biofilm.

**Fig. 3:**
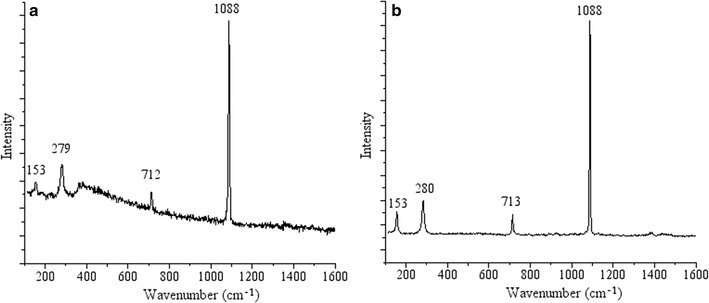
**a** Raman spectra of biomineralized calcium carbonate minerals in a *P. aeruginosa* biofilm. The highest Raman shift intensity appears at 1088 cm^−1^, indicating the presence of carbonate minerals. The primary peaks match those in a calcite standard spectrum (shown in** b**), indicating that the biomineralized deposits are calcite and not aragonite

To further verify the mineral composition of the inset material, we measured the mineralogy of these material with confocal Raman microscopy in a separate experiment. The spectra of precipitates within biofilms matched calcite Raman standard spectra in normal pressure and temperature (Fig. 3). The spectra showed the most notable peak at 1088 cm^−1^, which is caused by the symmetric stretching vibration of internal carbonate ion, the in-plane bending peak at 712 cm^−1^, and the lattice mode peak at 153 cm^−1^. Our results were also consistent with other findings investigating biomineralization induced calcite formation under natural or laboratory settings (Boquet et al. 1973; Morita 1980; Fujita et al. 2000). Aragonite is also a common calcium carbonate mineral produced by microbe. However, the Raman spectra of aragonite is featured with two in-plane bending peaks at 701 and 705 cm^−1^ and the lattice mode peak at 205 cm^−1^ (Wehrmeister et al. 2010), which was different from the spectra observed in the current experiment. Moreover, calcite has trigonal system crystal structure, and the carbonate ion appears as equilateral triangle, resulting in stable mineral structures. However, aragonite has orthorhombic system crystal structure, which is not stable and can be readily transformed to calcite. Different crystal structure yield distinct ion resonance states, which lead to distinct features in Raman spectra. Therefore, it is highly likely that calcite minerals were precipitated and inserted in the biofilm during biomineralization experiment.

These results clearly indicate that the combination of confocal laser scanning microscopy and confocal Raman microscopy provide an effective method to investigate the composition and morphology of mineral deposits induced by biomineralization within biofilms. It should be noted, both of these methods are nondestructive, and particularly are feasible for real time observation and quantitative analysis of mineral in situ precipitation processes within biofilms.

### Spatial patterns of calcium carbonate biomineralization

**Fig. 4:**
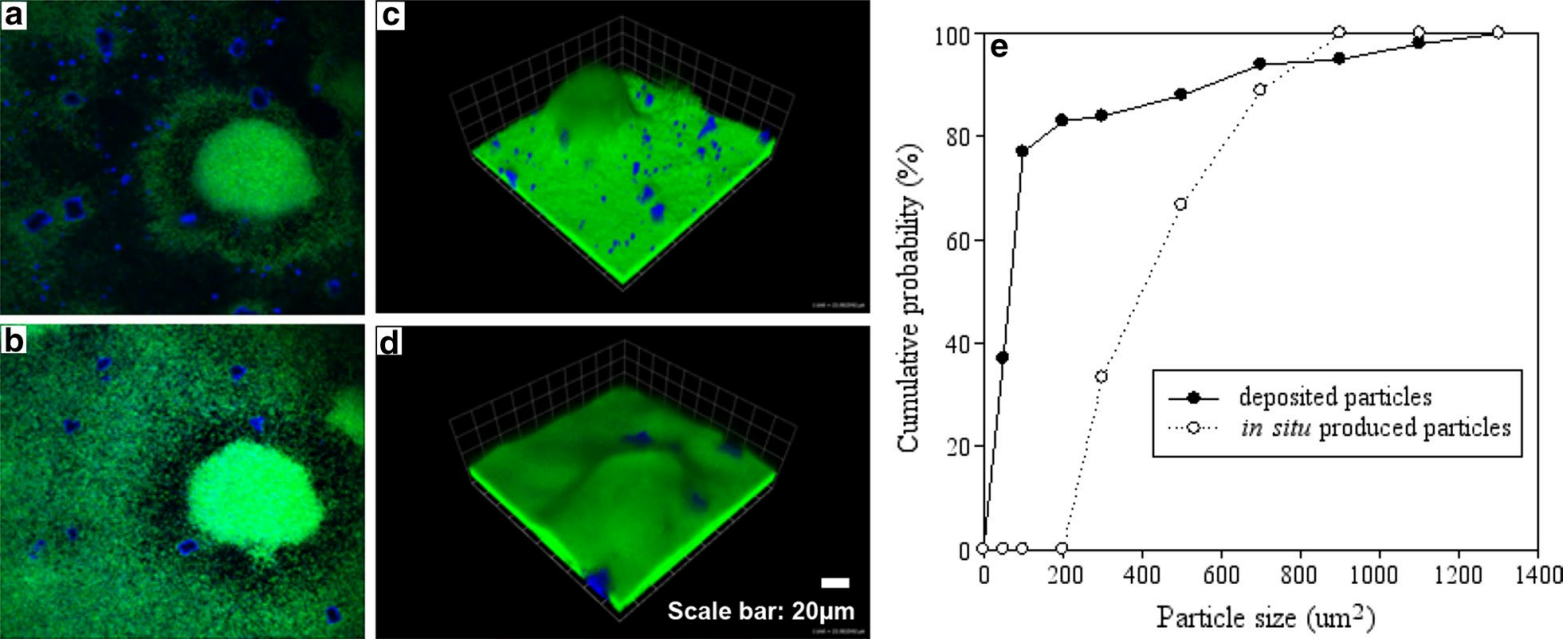
Biofilm traps precipitated fine calcite particles from bulk flow: **a**–**c** are under deposition mode and **d** is under suspension mode (control). **a** and **b** are xy planes of the same biofilm colony but at different depths. **a** is near bottom and **b** is near *top*. Mineral particle size distributions of **a** and **b** are plotted in **e**

It is also interesting to notice that the mineral deposits produced during the experiments showed distinct distribution patterns (Fig. 4): fine calcite particles that deposit at the surface of the biofilm and large granular calcite particles that root from the base of the flow cell. The differences in the sizes and distribution patterns of the mineral deposits are likely associated with the two production pathways of calcite: deposition of abiotically produced fine calcite particles and biomineralization induced large calcite granules. To verify this hypothesis, a flow cell with *P. aeruginosa* biofilm was inverted and the biomineralization experiment was performed under the same experimental conditions. As expected, the surface of the biofilm was free of fine calcite particles while large calcite granules grown from the base of the flow cell were still observed (Fig. 4d). Mineral particle size distribution further supported this hypothesis (Fig. 4e): the mineral deposits in the prior experiment was featured with large fractions of fine particles, with ~80% of the particles <100 µm^2^, while the mineral deposits in the latter experiment were all >200 µm^2^.

### Interactions of biofilm and mineral deposits

**Fig. 5:**
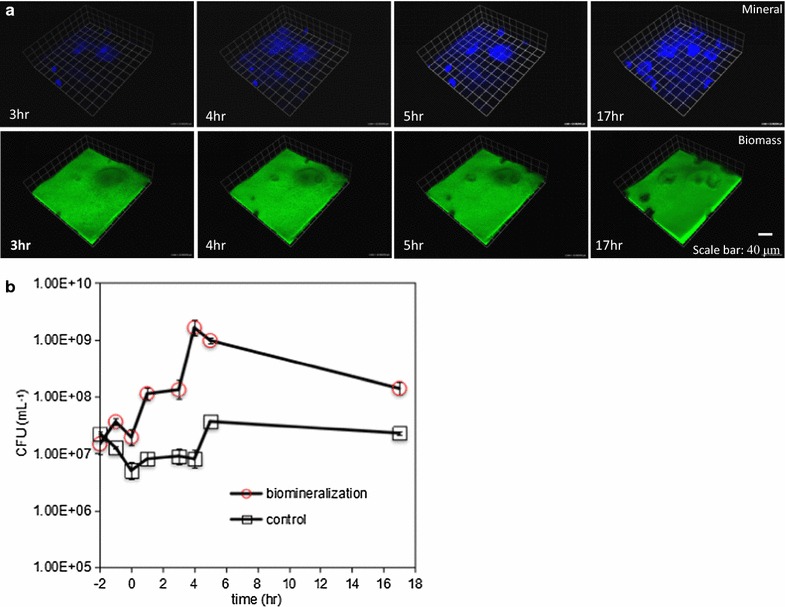
**a** Time series visualization of mineral growth and biofilm morphology. The same field of view was imaged at 3, 4, 5 and 17 h, the scale grid is 23 μm. **b** Detachment of cells from the biofilm during biomineralization. The cell concentration in the flow cell effluent was monitored for 2 h before biomineralization. Biomineralization medium were introduced at t = 0. Cell counts in the flow cell effluent significantly increased during biomineralization, indicating that biomineralization induced detachment of cells from the biofilm. Increased cell detachment was not observed in biofilms under control conditions (untreated biofilms)

The methods of laser reflection and fluorescence base on CLSM were used to observe and image calcium carbonate biomineralization and its spatial patterns within biofilms. We observed that the formation of inset calcite granules in the biofilm caused spatiotemporal collapse of the biofilm structure. The cells resided in biofilm were gradually replaced by the biomineral calcite formed, and the volume of biomass displaced was consistent with the volume of calcite formed (Fig. 5a). Under the invasion of growing biominerals, a dramatic detachment of cells in biofilm happened inevitably (Fig. 5b). Cell concentrations in the flow cell effluent were similar under both conditions and were at a magnitude of 10^7^ CFU ml^−1^. The number of CFU in the flow cell effluent significantly increased in biofilms under biomineralization treatment (Fig. 5b). The most prominent point of the cell concentration reached 10^9^ CFU ml^−1^ at 4 h, which was two orders of magnitude larger than the other points before introduction of biomineralization. The cell number detachment increasing was not observed in control experiments, which confirmed that the observed cell detachment from biofilms was yielded by biomineralization.

## Discussion

Using confocal microscopy, three dimensional real-time observation of calcium carbonate biomineralization in biofilms (PAO1-*gfp*) were achieved. On the feasible basis of flow cells, the confocal laser scanning microscopy provided fluorescence images of biofilms and reflection signal of mineral deposits, among these, the mushroom shape of biomass with green fluorescence was observed and the mineral deposits presented blue and irregular shape with clear outline. The shape of mineral deposits matched the vacancy of biofilms identically.

According to the different crystal structure, calcium carbonate can be mainly divided into three morphology of minerals, calcite, aragonite and vaterite (Wehrmeister et al. 2010). Even though confocal laser reflection achieve the observation of the distinction between mineral deposits and biofilms, the detailed mineralogy composition of the deposits could not be determined. We developed a combined method of confocal laser scanning microscopy and confocal Raman spectroscopy to delineate the morphology and identify the composition of the mineral deposits. The mineralogy of biomineralization products is calcite by spectra analysis of mineral deposits with confocal Raman spectroscopy. The spectra of calcite was distinct between aragonite by identifying Raman shift yielded by lattice vibration. The simultaneous obtainment of fluorescence (biomass) images and reflection signal (mineral deposits) makes it realize for a real time, in situ and nondestructive observation on the processes of biomineralization and mineral deposits producing.

A continuous controversy about how the microbial action influences carbonate precipitation in supersaturated environments and the function of microbial processes during the mineralization process has been existing for a long time. Previous studies reported that trapping of the abiotic particles was the primary route that accumulate mineral precipitates in the biofilm (Wuertz et al. 2003). Our results clearly showed that both trapping of abiotic calcite fine particles and biomineralization induced formation of calcite granules were two responsible mechanisms that *P. aeruginosa* biofilm accumulate minerals. Even though the in situ biomineralization and abiotic particles trapping occurred simultaneously in supersaturated environment, the abiotically formed calcite particles was confirmed to accumulate only on the surface of the biofilm. Moreover, the abiotically formed calcite fine particles are morphologically different from the in situ biomineral of calcite. In short, the saturation degree of calcium carbonate, inherent physiology of biofilm and surrounding environment the biofilm located are combined into the regulatory mechanism of microbial and abiotic precipitation processes. In addition, both of processes make contribution to microbial carbonate sequestration (Riding 2000).

Biofilm is well known to present varying and structured microenvironments as the synergistic effect of cell metabolism and delivery restriction (Stewart and Franklin 2008). We prospected to discover the same heterogeneity of in situ biomineralization as the heterogeneous calcite precipitation observed within biofilms. Based on prior theoretical predictions (Zhang and Klapper 2011), we also predicted that in situ biomineralization will appear on the biofilm surface primarily, yet, according to series of contrast and control experiments, microbial induced mineral deposits started to occur at the bottom of biofilms and grow upward, which is strikingly distinct patterns from the previous researches and relative hypothesis (Fig. 4). Hence, the observation results of biomineralization spatial distribution along with time we found which challenges usual common sense on MICP. Moreover, this study consider that the processes of microbial actions in situ dominate the biomineralization regulation, rather than ions release from bulk liquid in biofilms. Parallel result about spatial patterns of calcium-rich granules had also been published (Ren et al. 2008).

The mineral deposits was reported to influence the inner and external solution transportation patterns by altering the physiology of cells resident in biofilm (Hall-Stoodley et al. 2004; Uppuluri et al. 2010). Cell detachment variation measured by CFU counting methods demonstrated that formation of mineral deposits affect the metabolism of inner cells and change the settlement state (Fig. 5). The in situ biomineralization which leads to biofilm morphology change and structure collapse is treated as a crucial modulator for biofilm physiology.

Biofilm EPS was reported to promote biomineralization by providing ions and nucleation sites (Ercole et al. 2007). However, the functions of EPS in calcite in situ biomineralization is difficult to determine. Our results shown that there is no correlation between biomineralization patterns and the EPS density of biofilms (PAO1-*gfp*). Only some abiotic fine particles were trapped can’t establish a necessary connection of EPS and in situ biomineralization. Meanwhile, even though some researchers had found only active cells (biofilms) could induce mineral precipitation (Decho 2010; Zamarreno et al. 2009), mechanisms on how metabolism influences the processes of biomineralization are not well understood. Further research is needed to determine the accurate mechanism and develop effective experimental methods to investigate the mechanism that microbial processes that regulates biomineralization.

In general, this study provides a series of new experimental and visualization methods for investigating the in situ biomineralization in *P. aeruginosa* biofilms. The findings about spatial distribution patterns of biominerals would contribute to relevant further research of practical issues on MICP, such as microbial carbonate sequestration, lithiases with other serious complications and tracing biosignature record on Earth. The visualization methods can be used as a new tool combining with fluorescence imaging methods and widely applied in solving in situ microbial reaction kinetics and other biogeochemical processes.

## Additional file

**Additional file 1.** Nontoxic and biomineralization contrast experiment.

## Abbreviations

MICP: microbial induced carbonate precipitation
SI: saturation index
EPS: extra-cellular polymeric substance
PDMS: polydimethylsiloxane
GFP: green fluorescent protein
TSB: tryptic soy broth
OD: optical density
CLSM: confocal laser scanning microscopy
CCD: charged-coupled device
CFU: colony-forming unit
