# Supplementary material for: Experimental and visual research on the microbial induced carbonate precipitation by Pseudomonas aeruginosa
Source: AMB Express. 2017 Mar 9;7:57. doi: 10.1186/s13568-017-0358-5 (PMC5342990; doi:10.1186/s13568-017-0358-5)
Supplement: Supplementary file 1 — Additional file 1. Nontoxic and biomineralization contrast experiment. [file 13568_2017_358_MOESM1_ESM.doc]

**Supplementary Material**

**Nontoxic and biomineralization contrast experiment.**

For confirming the toxicity and precipitation status of different mineral medium with PAO1-*gfp* cells, the experiment was processed in three conditions. The PAO1-*gfp* cells were inoculated in the same way as described in manuscript previously in three divided flow cells. After that, we continuously pumped into nutrient which mixed three different mineral medium in the same concentration (15mM NaHCO_3_ only, 15mM CaCl_2_ only, 15mM NaHCO_3_ + 15mM CaCl_2_ )with 1% TSB medium separately. All these three flow cells were cultured for 3 days to grow mature at the same flow rate of 10ml/h. Then, the flow cell was visualized under Confocal microscopy with GFP and reflectance.

By the GFP signal, the results shown that after three days culture, PAO1-*gfp* cells grown healthy and formed biofilms in mushroom shape in all these three condition (Fig. S1 a, b and c). Even the number and size of these biofilms was nonuniform, we could confirm that mineral medium is non-toxic to PAO1-*gfp* cells. Furthermore, through reflectance signal, barely biomineralization precipitation was detected. This may be another evidence of the specific relationship between biomineralzation and matured biofilms.


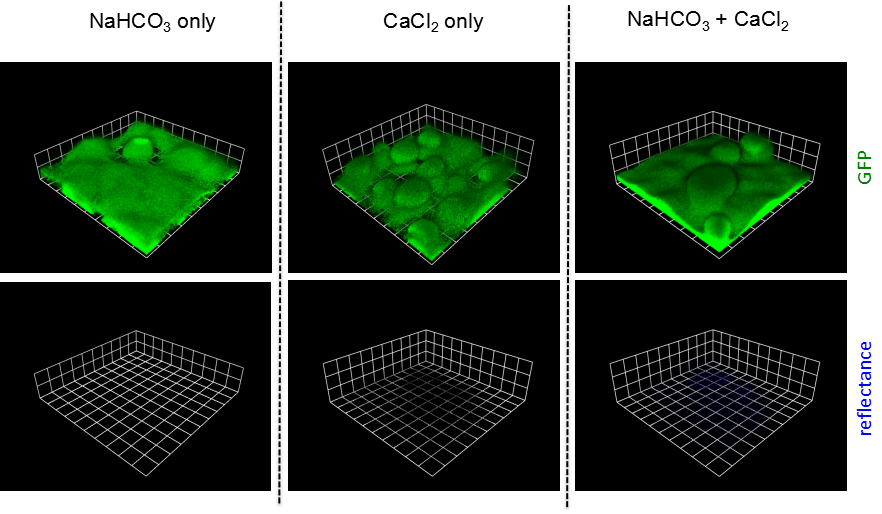


**Biomass**

**Scale bar: 20μm**

**a**

**b**

**c**

**Fig. S1** The GFP and reflectance signal in different mineral medium conditions after 3 days culture. (a) Only 15mM NaHCO_3_ with 1%TSB medium. (b) Only 15mM CaCl_2_ with 1% TSB medium. (c) 15mM NaHCO_3_ + 15mM CaCl_2_ with 1% TSB medium, the scale grid is 20 μm.
